# Cadmium Induces Transcription Independently of Intracellular Calcium Mobilization

**DOI:** 10.1371/journal.pone.0020542

**Published:** 2011-06-09

**Authors:** Brooke E. Tvermoes, Gary S. Bird, Jonathan H. Freedman

**Affiliations:** 1 Laboratory of Toxicology and Pharmacology, National Institute of Environmental Health Sciences, National Institutes of Health (NIH), Research Triangle Park, North Carolina, United States of America; 2 Laboratory of Signal Transduction, National Institute of Environmental Health Sciences, National Institutes of Health (NIH), Research Triangle Park, North Carolina, United States of America; 3 Nicholas School of the Environment, Duke University, Durham, North Carolina, United States of America; University of Hong Kong, Hong Kong

## Abstract

**Background:**

Exposure to cadmium is associated with human pathologies and altered gene expression. The molecular mechanisms by which cadmium affects transcription remain unclear. It has been proposed that cadmium activates transcription by altering intracellular calcium concentration ([Ca^2+^]_i_) and disrupting calcium-mediated intracellular signaling processes. This hypothesis is based on several studies that may be technically problematic; including the use of BAPTA chelators, BAPTA-based fluorescent sensors, and cytotoxic concentrations of metal.

**Methodology/Principal Finding:**

In the present report, the effects of cadmium on [Ca^2+^]_i_ under non-cytotoxic and cytotoxic conditions was monitored using the protein-based calcium sensor yellow cameleon (YC3.60), which was stably expressed in HEK293 cells. In HEK293 constitutively expressing YC3.60, this calcium sensor was found to be insensitive to cadmium. Exposing HEK293::YC3.60 cells to non-cytotoxic cadmium concentrations was sufficient to induce transcription of cadmium-responsive genes but did not affect [Ca^2+^]_i_ mobilization or increase steady-state mRNA levels of calcium-responsive genes. In contrast, exposure to cytotoxic concentrations of cadmium significantly reduced intracellular calcium stores and altered calcium-responsive gene expression.

**Conclusions/Significance:**

These data indicate that at low levels, cadmium induces transcription independently of intracellular calcium mobilization. The results also support a model whereby cytotoxic levels of cadmium activate calcium-responsive transcription as a general response to metal-induced intracellular damage and not via a specific mechanism. Thus, the modulation of intracellular calcium may not be a primary mechanism by which cadmium regulates transcription.

## Introduction

The transition metal cadmium is a persistent environmental toxicant. Diet, occupation, and smoking are the primary routes of cadmium exposure to the public. Exposure to this metal is associated with numerous human pathologies including kidney dysfunction, osteoporosis, respiratory ailments, and birth defects [1], [2], [3], [4]. In addition, cadmium is classified as a Type I human carcinogen, based on animal studies and data indicating that occupational exposure leads to an increased risk of lung cancer [5]. The prevalence of cadmium-associated diseases is increasing and cadmium-induced pathologies are appearing at levels below current OSHA standards [6], [7], [8].

*In vivo* and *in vitro* exposure to low concentrations of cadmium (1–5 µM) initiates an adaptive response that ameliorates the metal-induced toxicity. Toxic effects are reduced by increasing the levels of multiple stress-response proteins [9], [10], [11]. Analysis of transcriptome data from multiple species indicates that cadmium exposure alters the expression of hundreds of genes [9], [12], [13], [14]. Bioinformatic analyses of cadmium-transcriptomes identify the expected metal-responsive and stress-response processes/pathways including p38, extracellular signal-regulated kinase (ERK), and Jun N-terminal kinase (JNK)/mitogen-activated protein kinase (MAPK) pathways. Other pathways have been identified however, that cannot be directly associated with metal detoxification or the repair of metal-induced damage. In addition, the transcription of hundreds of additional genes is affected at higher, cytotoxic cadmium concentrations. An analogous process is observed in HepG2 cells treated with physiological and toxicological concentrations of copper [15].

The ability of cadmium to affect the expression of hundreds of functionally unrelated genes can be attributed to its capacity to modulate the activity of multiple signal transduction pathways. Cadmium activates p38, ERK, and JNK/MAPK pathways [16]. Activation of MAPK pathways affects the transcription of genes involved in the stress-response, as well as growth and development. In addition to the MAPK pathway, cadmium influences the activities of p53, NRF2, protein Kinase C, casein kinase 2, and calcium/calmodulin-dependent kinase II (CaMK II) [17], [18], [19], [20]. Cadmium may also influence gene expression by affecting the levels of second messengers, such as reactive oxygen species, cAMP and calcium.

It has been suggested that cadmium-activation of ERK, p38, and JNK results in part from an elevation of intracellular calcium concentration ([Ca^2+^]_i_) [21], [22]. While several studies indicate that exposure to cadmium causes increased [Ca^2+^]_i_, the mechanism by which cadmium affects [Ca^2+^]_i_ remains poorly understood [18]. Several factors have made defining the precise effects of cadmium on [Ca^2+^]_i_ problematic. A major issue has been the use of the calcium chelator 1, 2-bis(o-aminophenoxy)ethane-N,N,N',N'-tetraacetic acid (BAPTA) and BAPTA-based fluorescent calcium indicators. The BAPTA-based indicators and chelators are able to bind cadmium with high affinity. BAPTA binds calcium with a K_d_ ∼0.2 µM, however it also binds cadmium, but with K_d_ ∼1 pM. In addition, the fluorescent intensity of cadmium-bound Fura-2, a common BAPTA-based fluorescent dye used to monitor [Ca^2+^]_i_, is 70% greater for the cadmium-bound form compared to the calcium-bound form [23]. A second confounding factor is the use of cytotoxic concentrations of cadmium. LD_50_s for cadmium in mammalian cells are <10 µM, while studies examining the effects of cadmium on [Ca^2+^]_i_ routinely expose cells to concentrations of metal in far excess of this level [18], [21], [24]. Thus, there is a need to better understand the relationship between cadmium exposure, calcium mobilization, and the subsequent effect on transcription.

In the current report, the effect of cadmium on [Ca^2+^]_i_ is examined using the protein-based calcium sensor, yellow cameleon (YC)3.60, which is constitutively expressed in HEK293 cells (HEK293::YC3.60) [25]. The yellow cameleon does not respond to changes in intracellular cadmium concentration ([Cd^2+^]_i_). Exposing HEK293 cells to 1 µM cadmium for 4 h was sufficient to induce transcription of several cadmium-responsive genes, but did not affect cell viability, intracellular calcium levels, or the transcriptional activity of calcium-responsive genes. In contrast, exposure to cytotoxic levels of cadmium, 30 µM for 4 h, significantly decreased cell viability, reduced endoplasmic reticulum (ER) calcium stores, and significantly altered the transcriptional activity of calcium-responsive genes. These data indicate that non-cytotoxic concentrations of cadmium induce transcription independently of intracellular calcium mobilization. Furthermore, only when cells are exposed to cytotoxic cadmium concentrations is calcium mobilized from ER pools.

## Results

### Cadmium-inducible transcription in HEK293::YC3.60 cells

**Figure 1:**
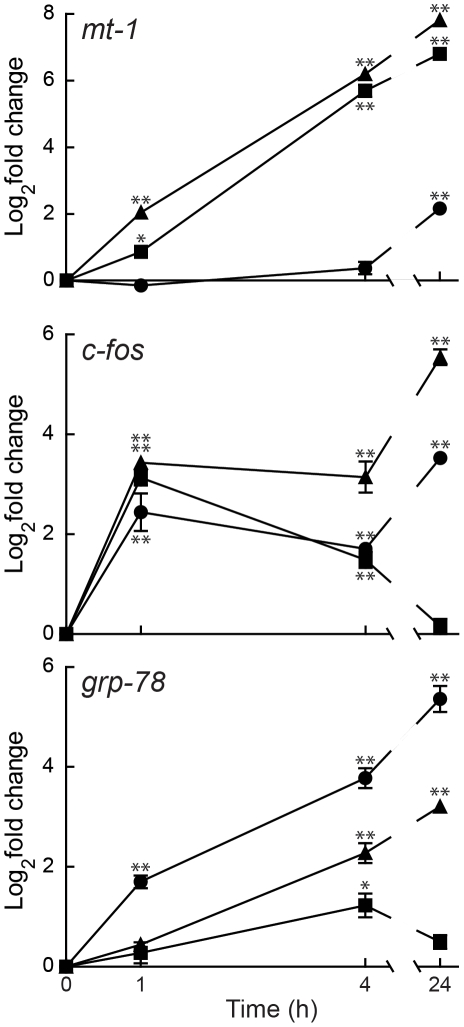
Effects of cadmium and thapsigargin on transcription. Total RNA was isolated from HEK293::YC3.60 cells exposed to either 1 µM (*square*) or 30 µM (*triangle*) cadmium, or 2 µM thapsigargin (*circle*) for 1, 4, or 24 h. Steady-state mRNA levels of *mt-1, c-fos,* and *grp-78* were measured using qRT-PCR. All measurements were normalized to mRNA levels of actin. Fold change was normalized to mRNA levels observed in control cells. Results were mean log_2_fold change ± SEM (*n* = 3) and were analyzed by one-way ANOVA followed by Dunnett's post-test; single (*) and double (**) asterisks indicate significant differences from controls at p<0.05 and p<0.001, respectively.

qRT-PCR of three well-characterized cadmium-inducible genes: *mt-1* (metallothionein-1), *c-fos*, and *grp-78* (78-kDa glucose-regulated protein/HSPA5) was used to quantify the transcriptional response of HEK293::YC3.60 cells exposed to cadmium or with altered [Ca^2+^]_i_. Exposure of HEK293::YC3.60 cells to 1 or 30 µM cadmium resulted in a rapid and significant increase in *mt-1* mRNA levels (Fig. 1). This observation was consistent with previous studies where cadmium exposure produced an increase in the steady-state levels of *mt-1* mRNA [11], [18]. Treatment with thapsigargin also caused an increase in *mt-1* mRNA, but only following a 24 h exposure. Thapsigargin is a potent inhibitor of calcium ATPases of the endoplasmic reticulum (ER), leading to a depletion of ER calcium and a concurrent increase in [Ca^2+^]_i_ [26], [27].

*c-fos* mRNA levels significantly increased following 1 h cadmium and thapsigargin exposures (Fig. 1). Longer exposures to 1 µM cadmium resulted in a gradual decrease in the steady-state level of *c-fos* mRNA, where at 24 h the mRNA level was not significantly different from control cells. The elevated levels of *c-fos* mRNA following 24 h exposures to 30 µM cadmium and thapsigargin may be the result of non-specific stress-responses (see below).

The steady-state level of *grp-78* mRNA increased in response to 1 and 30 µM cadmium and thapsigargin (Fig. 1). Thapsigargin had the fastest and largest effect on *grp-78* mRNA levels, followed by 30 µM cadmium. Similar to *c-fos*, exposure to 1 µM cadmium caused a transient elevation in *grp-78* which returned to baseline by 24 h.

The effect of cadmium and altered [Ca^2+^]_i_ following thapsigargin on the steady-state levels of *mt-1*, *c-fos*, and *grp-78* mRNAs in HEK293::YC3.60 cells were similar to those reported in previous studies [28]. These data indicated that this cell line would be an appropriate system to investigate mechanisms of cadmium-induced transcription and a potential contributory role of calcium.

### Intracellular cadmium accumulation and cell viability

**Figure 2:**
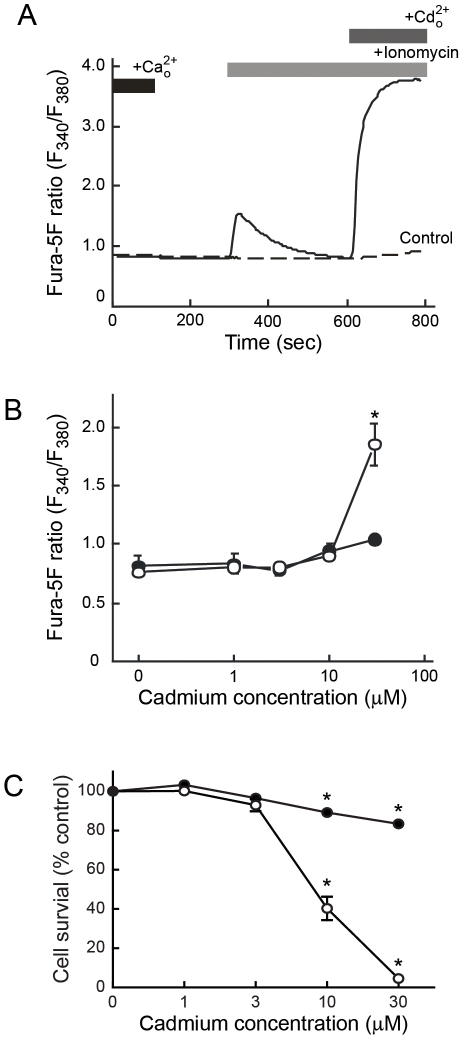
Cadmium uptake and cell viability in HEK 293::YC3.60 cells. *A*, Fura-5F loaded cells were incubated with ionomycin for 10 min to deplete intracellular calcium stores and then 2 mM cadmium in calcium-free HBSS was added to the medium (*solid line*). In the experiment represented by the *dashed line*, similar conditions were used except cells were not exposed to ionomycin prior to cadmium addition. Traces are representative of typical responses observed in at least three independent experiments. *B*, HEK293::YC3.60 cells were exposed to 0, 1, 3, 10, and 30 µM cadmium for 4 (*closed circle*) and 24 (*open circle*) h. Following metal exposure, cells were incubated with fura-5F and then fluorescence ratios were determined. Asterisks indicate a significant (p<0.001) difference between control and cadmium exposed groups. Data were analyzed by two-way ANOVA followed by Tukey's post-test. Asterisks indicate a significant (p<0.001) difference between control and cadmium exposed groups. *C*, Cell viability of HEK293::YC3.60 cells exposed to 0, 1, 3, 10, and 30 µM cadmium for 4 (*closed circle*) and 24 (*open circle*) h. Data were expressed as the mean ± SEM and were analyzed by one-way ANOVA followed by Dunnett's post-test. Asterisks indicate significant (*p*<0.001) difference from control and cadmium exposed groups.

Cellular uptake of cadmium in HEK293::YC3.60 cells was monitored using fura-5F, which binds cadmium with high affinity [23]. Prior to cadmium exposure, intracellular calcium stores were depleted by incubating cells for 5 min with 10 µM ionomycin, in calcium-free HBSS. This would ensure that changes in fluorescence ratios represented cadmium influx rather than changes in [Ca^2+^]_i_. The addition of 2 mM cadmium resulted in a rapid and significant increase in the fluorescence ratio (Fig. 2A). This indicated that cadmium entered the cells and that fura-5F was capable of detecting changes in [Cd^2+^]_i_.

To assess intracellular cadmium concentration ([Cd^2+^]_i_) levels in chronically treated cells, cells were exposed to various concentrations of cadmium for either 4 or 24 h and then loaded with fura-5F (Fig. 2B). Cadmium accumulation was both time- and concentration-dependent. The fura-5F fluorescence ratio showed the largest increase in cells exposed to 30 µM cadmium for 24 h.

Alterations in [Ca^2+^]_i_ occur during cadmium-induced cell death [21], [24], [29], [30], [31], [32], [33]. Therefore, the effect of cadmium on HEK293::YC3.60 cell viability was assessed (Fig. 2C). Exposure to 1 or 3 µM cadmium for 4 h or 24 h did not significantly affect the number of viable cells. These conditions were defined as non-cytotoxic cadmium concentrations. In contrast, exposure to higher cadmium concentrations for either 4 or 24 h significantly reduced cell viability compared to control cells and were therefore defined as cytotoxic cadmium concentrations. This suggests that changes in [Ca^2+^]_i_ at 10 or 30 µM cadmium may be a consequence of on-going cell death and not a direct activity of cadmium to induce intracellular calcium release.

### Effect of cadmium on YC3.60 expressed in HEK293 cells

**Figure 3:**
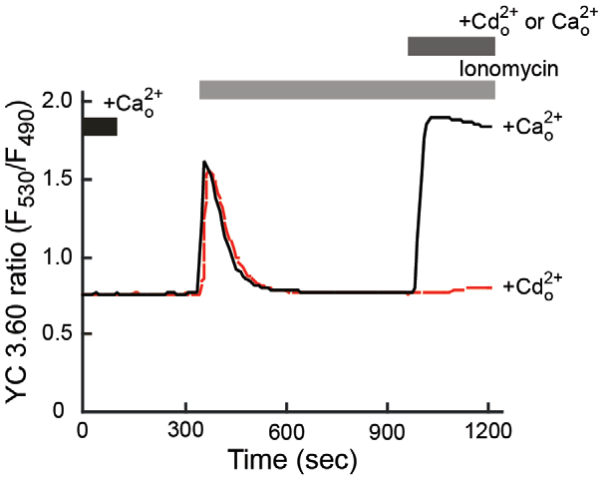
Effect of cadmium on YC3.60 fluorescence in HEK293::YC3.60 cells. Traces represent changes in YC3.60 fluorescence ratios following ionomycin exposure in calcium-free HBSS. At ∼15 min, the medium was supplemented with either 2 mM calcium (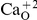, *black line*) or 2 mM cadmium (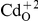, *red line*). Traces are representative of typical responses observed in four independent experiments.

The ability of YC3.60, a protein-based calcium ion sensor, to detect changes in [Cd^2+^]_i_ was assessed in HEK293::YC3.60 cells. Figure 3 presents a time course of a typical biphasic calcium response in HEK293 cells expressing YC3.60 in calcium-free media. Similar to studies using fura-5F loaded HEK293::YC3.60 cells (Fig. 2A), the addition of ionomycin produced a transient increase in [Ca^2+^]_i_. In addition, supplementing the medium with external calcium produced an influx of calcium, as indicated by a rapid and sustained increase in the fluorescence ratio (Fig. 3). In contrast to studies using fura-5F-loaded cells, the addition of 2 mM cadmium following ionomycin treatment did not affect the fluorescence ratio. These results demonstrated that YC3.60 could distinguish between calcium and cadmium ions; thus it would be a useful tool to measure the effects of cadmium on [Ca^2+^]_i_.

### Effect of cadmium on ER calcium stores and calcium uptake

**Figure 4:**
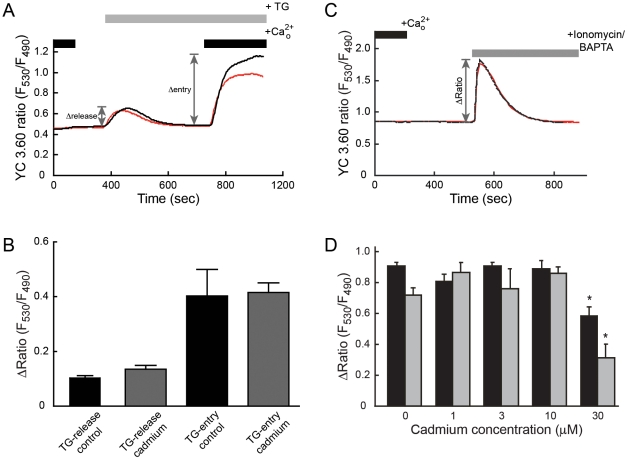
*panel A*, Traces represent [Ca^2+^]_i_ measured in control HEK293::YC3.60 cells (*black line*) or cells following exposure to 1 µM cadmium for 4 hr (*red line*). The traces are representative of typical responses observed in at least three independent experiments. *panel B,* Means of the peak values in thapsigargin response following exposure to 1 µM cadmium for 4 hr (*gray bar*) or non-cadmium treated (*black bar*). Data were expressed as the mean ± SEM and were analyzed by an unpaired *t-test*. There were no significant differences between control and the cadmium exposed groups. *panel C*, Traces represent [Ca^2+^]_i_ measured in HEK 293::YC3.60 cells under control conditions (*black line*) or following a 4 h exposure to 1 µM cadmium (*red line*). Following incubation with cadmium, cells were treated with an ionomycin-BAPTA solution in calcium-free HBSS. The traces were representative of typical responses observed in at least three independent experiments. *panel D*, Mean peak values of ionomycin responses in HEK293::YC3.60 cells following exposure to 0, 1, 3, 10, and 30 µM cadmium for 4 h (*black bar*) or 24 h (*gray bar*). Data were expressed as the mean ± SEM and were analyzed by one-way ANOVA followed by Dunnett's post-test. Asterisks (*) indicate significant difference (p<0.001) between control and cadmium exposed groups.

It has been proposed that cadmium activates transcription by depleting ER calcium stores to alter [Ca^2+^]_i_ [34]. To determine if non-cytotoxic concentrations of cadmium had an effect on calcium homeostasis or ER calcium stores, HEK293::YC3.60 cells were treated with the SERCA pump inhibitor thapsigargin prior to cadmium addition. Treating HEK293::YC3.60 cells with thapsigargin resulted in a typical biphasic change in [Ca^2+^]_i_, which represented depletion of ER calcium stores and the re-entry of calcium across the plasma membrane by the activation of store-operated calcium entry (SOCE) (Fig. 4A) [35], [36]. The biphasic calcium response induced by thapsigargin allowed several aspects of calcium homeostasis to be investigated: (i) thapsigargin-mediated inhibition of SERCA pump activity; (ii) proper operation of plasma membrane calcium-ATPases; and (iii) the activation mechanism and permeation properties of SOCE [37]. To determine if cadmium exposure altered the activity of these homeostatic processes, the thapsigargin-induced biphasic calcium response in cells exposed to 1 µM cadmium for 4 h was compared to control cells. The biphasic calcium response in HEK293::YC3.60 cells exposed to 1 µM cadmium was not significantly different from control cells (Fig. 4B). This indicated that exposure to cadmium at a concentration sufficient to induce transcription did not disrupt calcium homeostasis.

Any decrease in ER calcium store content would likely be reflected in a diminished thapsigargin-induced [Ca^2+^]_i_ peak. Exposure to cadmium however, did not significantly affect the thapsigargin-induced peak, which suggested that cadmium did not deplete ER calcium stores (Fig. 4B).

To further investigate the effect of cadmium on ER calcium stores, an ionomycin depletion protocol was utilized [38]. In the absence of extracellular calcium, ionomycin (10 µM) selectively triggers a rapid release of ER calcium stores [39]. The height of the ionomycin-induced [Ca^2+^]_i_ peak is approximately proportional to the ER calcium content (Fig. 4C). By comparing peak heights, the effects of various concentrations and exposure times of cadmium on ER calcium stores were determined. Following 4 or 24 h exposures, concentrations below 30 µM cadmium did not significantly affect ER calcium stores. A decrease of ER calcium store content was observed, however, in cells treated with 30 µM cadmium (Fig. 4D). The levels of ER calcium stores were reduced by 36% and 57% following 4 and 24 h exposures, respectively.

These data suggest that low concentrations of cadmium (1–10 µM), which induced changes in gene transcription (Fig. 1), did not interfere with calcium signaling mechanisms nor deplete ER calcium stores.

### Effects of cadmium and thapsigargin on cAMP/Ca^2+^ responsive gene expression

**Figure 5:**
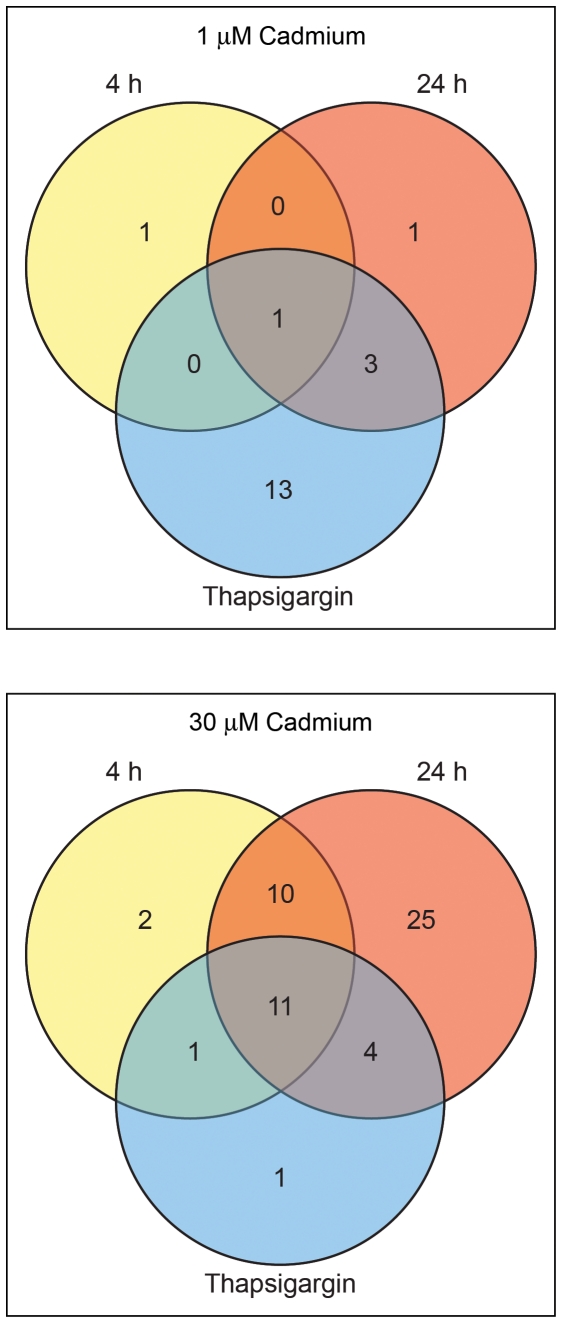
Venn diagram illustrating genes whose steady-state levels of expression change in HEK293::YC3.60 cells following exposure to 1 or 30 µM cadmium for 4 or 24 h, or 2 µM thapsigargin for 4 h. The identity and description of the eleven common genes are presented in Table 4.

PCR arrays were used to assess the ability of cadmium to affect the steady-state levels of mRNAs encoded by 84 cAMP/calcium -responsive target genes. Consistent with the [Ca^2+^]_i_ measurements (Fig. 4), 1 µM cadmium did not significantly affect the transcription of the majority of the cAMP/calcium-responsive genes (Tables 1 and 2). Exposure to 30 µM cadmium for 4 or 24 h, however, significantly affected the mRNA levels of many of the cAMP/calcium -responsive target genes. This response may be the result of 30 µM cadmium depleting ER calcium stores thus increasing [Ca^2+^]_i_ (Fig. 4D), or a consequence of cadmium-mediated cell death (Fig. 2C), which also alters [Ca^2+^]_i_. To determine whether the ability of cadmium to induce transcription was due to changes in [Ca^2+^]_i_, the patterns of cadmium-responsive gene expression were compared to those produced following thapsigargin exposure (Table 3). Exposure to 1 µM cadmium resulted in the differential expression of four genes that were also affected by thapsigargin exposure (Fig. 5). For these genes (TNF, FOS, PTGS2, and ERG1) however, cadmium exposure caused a significant decrease in the steady-state level of mRNA, whereas thapsigargin exposure produced a significant increase in mRNA levels.

**Table 1:** SuperArray analysis of cAMP/calcium signaling in cells exposed to cadmium for 4 h.

Gene Name	Fold Change	*p* value
**1 µM Cadmium (4 h)**		
PENK	2.4512	0.023029
TNF	-3.4233	0.012398
**30 µM Cadmium (4 h)**		
FGF6	24.6539	0.005357
DDIT3	9.0099	0.000048
DUSP1	6.6408	0.000159
PENK	4.664	0.000512
ATF3	4.6518	0.000101
PPP1R15A	4.6185	0.000054
INHBA	4.3349	0.000586
CNN1	4.2822	0.002495
EGR1	3.9973	0.016708
AREG	3.3761	0.002253
HSPA5	3.0578	0.000963
FOSB	3.0299	0.001182
TACR1	2.8573	0.005705
GEM	2.7639	0.000113
SCG2	2.6789	0.029449
EGR2	2.3043	0.018078
FOS	2.1596	0.033391
PTGS2	2.1378	0.056764
JUND	2.1214	0.001347
TNF	-19.1092	0.000215
JUNB	-2.3095	0.000264
ADRB1	-2.1577	0.000071
PER1	-2.0993	0.00208
PLN	-2.0285	0.09175

**Table 2:** SuperArray analysis of cAMP/calcium signaling in cells exposed to cadmium for 24 h.

Gene Name	Fold Change	*p* value
**1 µM Cadmium (24 h)**		
TNF	-13.0436	0.000352
FOS	-3.7234	0.003562
PTGS2	-3.3062	0.023548
THBS1	-2.3139	0.005863
EGR1	-2.0611	0.020815
**30 µM Cadmium (24 h)**		
FOSB	1994.706	0.000001
CHGA	1270.4422	0.000002
EGR1	1247.6159	0.000005
FGF6	740.5477	0.000597
CGA	226.4632	0.000004
TH	85.4584	0.00002
EGR2	76.2662	0.000199
PPP1R15A	70.6985	0.000005
DDIT3	61.3558	0.000042
AREG	57.1957	0.000005
IL6	51.4587	0.000126
FOS	51.0661	0.000006
S100A6	46.5164	0.000114
TACR1	43.5103	0.000079
GEM	42.7737	0.000025
JUND	28.2554	0.006534
PRL	27.6541	0.000204
CNN1	26.1911	0.000112
INHBA	25.5051	0.000085
POU1F1	20.8117	0.000247
CREM	20.704	0.000779
PTGS2	20.6576	0.000475
HSPA5	20.0538	0.000024
CALB1	19.4826	0.003432
JUNB	16.0708	0.000079
SCG2	14.004	0.001741
GIPR	12.922	0.00001
CALB2	12.3142	0.000762
SGK1	10.5842	0.000023
NR4A2	9.6651	0.00006
THBS1	8.3133	0.000252
SSTR2	7.538	0.001687
MAF	7.0063	0.001082
NOS2A	6.3728	0.002331
HK2	5.6116	0.000267
TNF	5.5566	0.006301
NPY	4.7459	0.000043
DUSP1	4.5155	0.000003
PENK	4.1311	0.005164
CTF1	2.9341	0.021035
BCL2	2.8517	0.000331
CCNA1	2.7945	0.015029
CALR	2.5418	0.003024
ATF3	2.5353	0.001326
PLAT	2.4699	0.046362
PMAIP1	2.3666	0.002755
CREB1	-5.9272	0.052032
NCAM1	-2.4919	0.041313
LDHA	-2.059	0.010214
PRKAR1A	-2.0369	0.008681

**Table 3:** SuperArray analysis of cAMP/calcium signaling in cells exposed to thapsigargin.

Gene Name	Fold Change	*p* value
2 µM Thapsigargin (4 h)		
DDIT3	21.2583	0.00017
PTGS2	15.4315	0.011724
HSPA5	7.9023	0.00148
INHBA	7.8966	0.004143
NR4A2	5.6134	0.004956
IL6	5.0238	0.014326
FOSB	4.9708	0.079038
PPP1R15A	4.3266	0.001643
GEM	4.2286	0.001231
EGR1	4.1209	0.048005
PCK2	3.8763	0.001097
ATF3	3.5312	0.000724
CGA	3.242	0.020191
HK2	2.5842	0.133179
PER1	2.541	0.018747
FOS	2.4214	0.003781
TNF	2.2131	0.048366

For 30 µM cadmium, the expression of eleven genes was significantly affected among the three exposure conditions: 4 and 24 h 30 µM cadmium and 4 h 2 µM thapsigargin (Fig. 5, Table 4). Gene Ontology analysis showed that these genes were involved in apoptosis, differentiation, and mitogenesis. Fifteen of the seventeen genes induced by thapsigargin were up-regulated in response to a 24 h exposure to 30 µM cadmium. The majority of the genes affected by cadmium, however, were not differentially expressed following thapsigargin exposure. In addition, the expression of TNF and PER1 was suppressed by cadmium exposure, but increased following thapsigargin exposure. Together, these results suggested that exposure to cytotoxic concentrations of cadmium affected transcription via a mechanism that may be independent of changes in [Ca^2+^]_i_.

**Table 4:** Genes affected by both cadmium and thapsigargin exposures.

Gene	Name	Description^a^
DDIT3	DNA damage-inducible transcript 3	Transcription factor that promotes cell death during ER stress
PTGS2	Prostaglandin-endoperoxide synthase 2	Responsible for the prostanoid biosynthesis involved in inflammation and mitogenesis
HSPA5	Heat shock 70kDa protein 5	Involved in the folding and assembly of proteins in the ER during stress
TNF	Tumor necrosis factor	Multifunctional proinflammatory cytokine that is mainly secreted by macrophages
INHBA	Inhibin beta A subunit	Member of the transforming growth factor-beta superfamily that may acts as both a growth/differentiation factor and a hormone
FOSB	FBJ murine osteosarcoma viral oncogene homolog B	Part of the transcription factor complex AP-1 and regulates cell proliferation, differentiation, and transformation
PPP1R15A	Protein phosphatase 1, regulatory (inhibitor) subunit 15A	Helps mediate apoptosis following stressful conditions
GEM	GTP binding protein over-expressed in skeletal musclehttp://www.genenames.org/data/hgnc_data.php?hgnc_id=4234	Belongs to the RAD/GEM family of GTP-binding proteins, could play a role in receptor-mediated signal transduction
EGR1	Early growth response 1http://www.genenames.org/data/hgnc_data.php?hgnc_id=3238	Transcriptional regulator of genes required for differentiation and mitogenesis
ATF3	Activating transcription factor 3	Member of the CREB protein family and mediates pro-apoptotic effects of p38
FOS	FBJ murine osteosarcoma viral oncogene homolog	Part of the transcription factor complex AP-1 and regulates cell proliferation, differentiation, and transformation

a Name and description were determined using GeneCards [59].

## Discussion

Cadmium is a well-known toxicant that is continuously introduced into the environment. Environmental exposure to cadmium is a substantial public health concern. Recent studies suggest that incidences of cadmium-associated disease are escalating in populations exposed to low levels of cadmium [6], [8], [40], [41]. To more fully understand the relationship between cadmium and disease, it is imperative to understand the mechanism of cadmium-responsive transcription, under both adaptive and toxicological conditions. Toxicological effects at low-levels of exposure are prevented or repaired by altered expression of multiple stress-response proteins and their cognate genes. Alterations in gene expression have been observed in multiple systems at cadmium concentrations below those leading to measurable toxicological responses. *In vitro* exposure of HeLa or CCRF-CEM cells to non-toxic concentrations of cadmium affects the expression of ∼60 and 20 genes, respectively [42], [43]. Treatment of mice with non-toxic doses of cadmium causes the differential expression of 22 genes [44]. Likewise in *C. elegans*, ∼100 genes are differentially expressed following cadmium exposure under conditions that do not induce a general stress response [13].

At toxic concentrations of cadmium, the transcription of hundreds of genes is affected. Among the hundreds of cadmium-responsive genes, many of the cognate regulatory pathways have been identified. These pathways include MAPK, p53, NRF2, Protein Kinase C, casein kinase 2, and CaMK II [17], [18], [19], [20]. Although regulatory pathways have been identified, the molecular mechanisms by which cadmium initially activates these pathways to elicit specific transcriptional changes have not been defined.

One hypothesis that addresses how cadmium activates intracellular signaling pathways proposes that the metal modulates the level of [Ca^2+^]_i_ [34]. Thus, calcium could be viewed as a second messenger that mediates cadmium-responsive transcription. While several mechanisms have been proposed by which cadmium may alter [Ca^2+^]_i_, the effects of cadmium on calcium signaling remain ambiguous. This ambiguity may be a consequence of technical approaches traditionally used to investigate calcium-mediated signaling processes. Specifically, there are potential problems in the interpretation of data from studies in which BAPTA or BAPTA-based fluorescent calcium indicators are used when examining the consequences of cadmium exposure on [Ca^2+^]_i_. Cadmium binds to these compounds with a >1000-fold higher affinity and can produce higher fluorescence than calcium making the interpretation of this data problematic [23]. A loss of a response during co-exposures of BAPTA with cadmium could be due to decreases in the effective cadmium concentrations, rather than effects on [Ca^2+^]_i_. Likewise, an increase in fluorescence in fura-loaded cells following exposure to cadmium could be due to the binding of cadmium to the dye, rather than a release of intracellular calcium from storage. To circumvent this problem, a protein-based calcium indicator, yellow cameleon 3.60 was used in the current studies. In HEK293::YC3.60 cells, cadmium exposure did not elicit a change in YC3.60 fluorescence (Fig. 3). Under similar experimental conditions however, cadmium produced significant increases in fura-5F fluorescence ratios (Fig. 2). This indicates that fura-5F and potentially other BAPTA-based fluorescent dyes can be used to measure [Cd^2+^]_i_, but are not appropriate when measuring the effects of cadmium on [Ca^2+^]_i_.

To assess the effects of cadmium on intracellular calcium homeostasis, HEK293 cells that stably expressed YC3.60 were used. YC3.60 provides a direct measure of [Ca^2+^]_i_ without interference from cadmium (Fig. 3). A second consideration in the current experimental design is the use of non-cytotoxic concentrations of metal. Exposing HEK293::YC3.60 cells to 1 µM cadmium for 4 h was sufficient to increase steady-state mRNA levels of three well-characterized cadmium-responsive genes (Fig. 1). In addition, exposure to 1 µM cadmium for 4 or 24 h did not produce any significant toxicological responses (Fig. 2C). Based on these results, subsequent calcium homeostasis and signaling experiments were performed using HEK293::YC3.60 cells exposed to 1 µM cadmium. To replicate previous studies and gain an understanding of how cytotoxic levels of cadmium affect [Ca^2+^]_i_, cells were also exposed for 4 and 24 h to 30 µM cadmium, which is approximately three-times the 24 h LC_50_ for this cell line.

Using this experimental design, low-dose cadmium exposures did not interfere with calcium homeostasis nor deplete ER calcium store content (Fig. 4). Only high concentrations of cadmium (30 µM) depleted ER calcium stores. This is similar to that reported by Biagioli *et al*., who observed a significant depletion of ER calcium stores in NIH 3T3 cells treated with 15 µM cadmium for 12 h [34]. The reported LC_50_s for cadmium in 3T3 cells range from 1–5 µM [45]. These results suggest that as cells succumb to metal toxicity, calcium is released from intracellular stores.

Cadmium exposure increases the activity of MAPK and CaMK II regulated pathways [16], [17], [20], [21], [46]. Since MAPKs and CaMK II are considered integrators of calcium signaling, the effect of cadmium on the expression of calcium responsive genes was investigated using cAMP/calcium signaling focused arrays. Exposure of HEK293::YC3.60 cells to non-cytotoxic levels of cadmium, 1 µM for 4 or 24 h, did not affect the expression of a significant number of genes. One gene was commonly affected by both non-cytotoxic cadmium conditions and thapsigargin-induced intracellular calcium release. Following 24 h exposure to1 µM cadmium, an additional three genes were affected by both cadmium and thapsigargin. However, among the commonly affected genes; TNF, FOS and EGR1; cadmium caused a significant decrease in their steady-state mRNA levels while an increase in [Ca^2+^]_i_ had the opposite effect (Table 2). These results are consistent with the lack of a significant effect on [Ca^2+^]_i_ in cells exposed to low concentrations of cadmium (Fig. 4). These metal concentrations are associated with adaptive responses and do not deplete ER calcium stores nor interfere with intracellular calcium signaling. Thus under these conditions calcium does not function as a second messenger mediating cadmium-responsive transcription.

Exposure to cytotoxic levels of cadmium affected the steady-state mRNA levels of ∼60% of the genes, in contrast to non-cytotoxic conditions that affected ∼2% (Fig. 5, Table 1 and 2). These results are similar to previous studies demonstrating concentration-dependent increases in the number of affected genes when cells are exposed to environmental toxicants; i.e., as the concentration of toxicant increases from adaptive to cytotoxic, there is a concomitant increase in the number of genes whose steady-state level of expression change. This was observed in HepG2 cells exposed to copper; where at physiological copper concentrations (200 µM) the expression of 30 genes was affected, but at toxicological concentrations (600 µM) the number of affected genes increased to 790 [15].

Exposure to 30 µM cadmium for 24 h affected the expression of 50 genes. This result was consistent with the [Ca^2+^]_i_ measurements in which 30 µM cadmium affected ER calcium stores (Fig. 4). The majority of the thapsigargin-inducible genes were also affected by 30 µM cadmium. However, three-times as many genes were affected by cadmium as thapsigargin, 17 vs. 53 (Fig. 5). In addition, the steady-state mRNA levels of TNF and PER1 increased in response to intracellular calcium release, but decreased following cadmium exposure. This suggests that the overlap among affected genes may be the result of a general activation of transcription by cadmium rather than a specific calcium-mediated effect.

Exposure to 30 µM cadmium caused a significant decrease in cell viability and depletion of ER calcium stores. At cytotoxic concentrations, calcium release may not be a specific cadmium-induced response; rather it could be a secondary or tertiary response, or non-specific affect. For example, cadmium exposure in rodents causes an increase in cAMP levels by increasing adenylate cyclase and decreasing cAMP phosphodiesterase activities, which ultimately leads to the activation of cAMP-dependent protein kinase regulated genes [47]. Similarly, the activation of DNA damage response is due to cadmium-induced DNA damage via oxidative stress and inhibition of DNA repair, and not a direct interaction between cadmium and p53 [48]. In addition, the activation/suppression of transcription could be a consequence of metal-induced membrane damage and cell death. As a consequence of the breakdown of intracellular structures, calcium would be released from membrane-bound intracellular stores. Cadmium-induced oxidative stress and lipid peroxidation occur within minutes of exposure to toxic concentrations of metal and prior to any measurable cytotoxicity [49], [50], [51]. Metal-induced damage could activate multiple processes. The activation of signaling proteins and cognate regulatory pathways would affect the expression of dozens of genes including the calcium/cAMP responsive genes on the array.

Low-level exposure to cadmium is relevant to human health as the general population is constantly exposed to low levels of this metal. Exposure to non-cytotoxic levels of cadmium is sufficient to affect gene expression, but does not alter calcium homeostasis. In addition, the transcription of calcium/cAMP responsive genes is unaffected by non-cytotoxic levels of cadmium. These data strongly suggest that cadmium-activated transcription is independent of intracellular calcium signaling. The results also support the hypothesis that at cytotoxic concentrations of cadmium, calcium-regulated signaling is affected as part of a general downstream response to cadmium-induced intracellular damage, and not a specific effect of cadmium on calcium homeostasis. They also suggest that further examination of the molecular mechanisms regulating cadmium-responsive transcription should be conducted at non-cytotoxic metal concentrations, which are environmentally relevant, and confirm that experimental reagents do not interact with cadmium.

## Materials and Methods

### Cell culture

HEK293 cells stably expressing the calcium sensitive protein yellow cameleon 3.60 (HEK293::YC3.60) were generated by transfecting HEK293 cells with YC3.60 cDNA using lipofectamine 2000 according to manufacturer's instructions (Invitrogen/Life Technologies, Carlsbad, CA). Details about the YC3.60 plasmid and its construction can be found in Nagai, *et al*. [25]. Following a 48 h recovery period, transfected cells were sorted on a FACSVantage SE Flow Cytometer (BD Bioscience, San Jose, CA) to select and enrich the cell population for cells expressing YC3.60. The sorting and enriching procedure was repeated three times to establish a population of cells homogeneously expressing YC3.60. Subsequently, HEK293::YC3.60 cells were maintained in culture at 37°C in a 5% CO_2_ atmosphere in Dulbecco's modified Eagle's medium supplemented with 2 mM glutamine, 10% fetal bovine serum, and 75 µg/ml G-418 (Invitrogen/Life Technologies). Cells stably expressing YC3.60 were used for all cell culture experiments.

### Cell viability assays

Sensitivity of HEK293::YC3.60 cells to increasing concentrations and exposure times to cadmium was determined by using the neutral red assay as previously described [52]. HEK293::YC3.60 cells were exposed to 0, 1, 3, 10, or 30 µM cadmium for 4 or 24 h. Quadruplicate experiments were performed at each concentration. Results are presented as a percentage of control values, mean ± standard mean error (SEM) (*n* = 4). Data were analyzed by one-way ANOVA followed by Dunnett's Multiple Comparison post-test.

### Isolation of total RNA and qRT-PCR

To assess the effects of cadmium and thapsigargin on transcription in HEK293::YC3.60 cells, steady-state levels of *mt-1*, *c-fos*, and *grp-78* mRNA were determined using quantitative real time PCR (qRT-PCR). Cells were treated with cadmium (1 or 30 µM) or thapsigargin (2 µM) for the last 1, 4, or 24 h of 48 h incubations. Total RNA was then isolated from both treated and control cells using RNeasy Mini Kits following manufacturer's instructions (Qiagen, Inc., Valencia, CA).

For qRT-PCR, total RNA from three independent experiments was isolated from treated and control cells. Each biological replicate was measured in triplicate by two-step qRT-PCR using SuperScript First-Strand Synthesis System for RT-PCR (Invitrogen) and SYBR Green (Qiagen) as previously described [53]. Fold changes in mRNA levels were calculated using the ΔΔCt method with β-actin as reference mRNA [54], [55]. Changes in gene expression for cadmium-treated cells were compared to the level of expression in non-treated cells. Changes in gene expression for thapsigargin-treated cells were compared to DMSO vehicle-treated cells. Results are presented as mean log_2_ (fold change) ± SEM. Sequences for primers used in the qRT-PCR are presented in Table S1.

### Measurements of intracellular calcium concentration

For [Ca^2+^]_i_ measurements, log-phase cells were transferred onto 30 mm round glass coverslips and allowed to attach in a small volume of medium for 4–6 h. Additional DMEM was then added and the cells incubated for 24–36 h before [Ca^2+^]_i_ measurements.

#### Fura-5F-based calcium measurements

Cells were loaded in the dark with fura-5F by incubating coverslips, mounted in a Teflon incubation chamber, with 1 µM fura-5F/AM in DMEM at 37°C for 25 min. Immediately before [Ca^2+^]_i_ measurements, cells were washed three times and incubated for 15–30 min at room temperature in HEPES-buffered salt solution (HBSS; 120 mM NaCl, 5.4 mM KCl, 0.8 mM MgCl_2_, 1.8 mM CaCl_2_, 10 mM glucose in 20 mM HEPES, pH 7.4). Teflon chambers were then mounted onto a Nikon TS-100 inverted microscope equipped with a 20X objective (0.75 NA). In experiments where cells were incubated in nominally calcium-free buffer, HBSS with no added CaCl_2_ was used. Fluorescence images were recorded and analyzed, as previously described [56]. Changes in intracellular calcium are represented by changes in the ratio of the fura-5F fluorescence at 340 nm to that at 380 nm (*F_340_/F_380_*). Ratio changes were obtained from multiple regions of interest (ROI), where each ROI represented an individual cell. Typically, 25 to 35 ROIs were monitored per experiment. Ratio values were corrected for autofluorescence, which was determined after treating cells with 10 µM ionomycin and 20 mM MnCl_2_ [37]. Because BAPTA-based calcium indicators bind cadmium with high affinity, fura-5F-loaded HEK293::YC3.60 cells were also used to monitor the accumulation of [Cd^2+^]_i_.

#### YC3.60-based calcium measurements

HEK293::YC3.60 cells were mounted in Teflon incubation chambers and maintained in HBSS. Chambers were mounted on a Zeiss LSM 510 confocal microscope equipped with a 20X objective (NA 0.8). The YC3.60 FRET signals were monitored by exciting at 458 nm and collecting emission images at 490 nm for CFP and 530 nm for YFP [57]. After correcting for background fluorescence, [Ca^2+^]_i_ levels were monitored by calculating the fluorescence ratio of YFP to CFP emissions (*F_530_/F_490_*). An increase in [Ca^2+^]_i_ was observed as an increase in the *F_530_/F_490_* ratio. Typically, fluorescence signals from 25–30 ROIs were monitored for a single experiment.

### Effects of cadmium on intracellular calcium pools and store-operated calcium entry

The status of intracellular calcium pools following exposure to 0, 1, 3, 10 or 30 µM cadmium for 4 or 24 h was assessed by exposing HEK293 cells to 10 µM ionomycin in the presence of 3 mM BAPTA [37]. Following incubation in cadmium containing buffer, cells were mounted in a Teflon incubation chamber and maintained in HBSS for ∼10 min before being treated with 10 µM ionomycin in the presence of 3 mM BAPTA [38]. The transient calcium response was indicative of the size of intracellular calcium pool and could be quantified by calculating the peak YC3.60 response. The peak YC3.60 response was defined by the maximal response minus the resting fluorescence ratio.

To investigate the effects of cadmium on SOCE, a “calcium re-addition” protocol was used in HEK293::YC3.60 cells treated with 2 µM thapsigargin [37]. In cases where the cadmium concentration was cytotoxic, YC3.60 fluorescence was monitored only in recognizably viable cells. All data were analyzed by one-way ANOVA followed by Dunnett's Multiple Comparison post-test.

### Human cAMP/calcium Signaling RT^2^ Profiler^TM^ PCR Array

Human cAMP/calcium Signaling RT^2^ Profiler^TM^ PCR Arrays (SABiosciences, Frederick, MD) were used to examine the effect of cadmium on calcium-responsive transcription in HEK293::YC3.60 cells. These arrays contain 84 genes that are reported to be responsive to changes in cAMP and calcium levels. The layout and description of the genes on the array are presented in Table S2.

Cells were grown and RNA was isolated as described above. The purity and quality of RNA was assessed using the RNA 6000 LabChip and Agilent 2100 Bioanalyzer (Agilent Technologies, Santa Clare, CA, USA). Procedures for qRT-PCR and array analysis were performed according to manufacturer's instructions. Array data was normalized to the average threshold cycle (*C_t_*) value of three housekeeping genes: β-2-microglobulin (B2M), hypoxanthine phosphoribosyltransferase (HPRT1), and ribosomal protein L13a (RPL13A). These genes were chosen for normalization because their *C_t_* values did not differ by more than one cycle among all of the samples and treatments. The average *C_t_* value for the three housekeeping genes did differ by more than one cycle in cells treated with 30 µM cadmium for 24 h.

To make a meaningful biological analysis of differentially expressed genes in cells treated with 30 µM cadmium for 24 h, a normalization factor was calculated for each of the three independent experiments (0.91, 0.88, and 0.89) so that the normalized expression ratio of the average *C_t_* value for each housekeeping gene was equal across compared samples (cadmium treated vs. control). This normalization factor was then used to appropriately scale the expression value for each of the 84 calcium specific genes within each array for cells treated with 30 µM cadmium for 24 h. These normalized expression values were then used to determine fold change. Data was then processed with SABiosciences web-based RT^2^ Profiler^TM^PCR Array Data Analysis to calculate *C_t_* and relative gene expression values according to the ΔΔ *C_t_* method [58]. A list of differentially expressed genes was identified using a Student's *t*-test. Only two-fold or greater changes in gene expression with a *p*<0.05 were considered significant.

## Supporting Information

Table S1 Sequences of primers used for qRT-PCR.
(DOCX)

Table S2 Functional gene grouping of human cAMP/calcium PCR Array.
(DOCX)
