# Supplementary material for: Cadmium Induces Transcription Independently of Intracellular Calcium Mobilization
Source: PLoS One. 2011 Jun 9;6(6):e20542. doi: 10.1371/journal.pone.0020542 (PMC3111418; doi:10.1371/journal.pone.0020542)
Supplement: Table S1 — Sequences of primers used for qRT-PCR. (DOCX) [file pone.0020542.s001.docx]

**Table S1**

Sequences of primers used for qRT-PCR.

| **Gene** | **Forward (5’ to 3’)** | **Reverse (5’ to 3’)** |
| --- | --- | --- |
| actin | GATATCGCTGCGCTGGTCGTC | ACGCAGCTCATTGTAGAAGGTGTGG |
| mt-1 | GCAAATGCAAAGAGTGCAAA | CAGCTGCACTTCTCTGATGC |
| c-fos | GATACACTCCAAGCGGAGACAGA | GTGAGCTGCCAGGATGAACT |
| grp-78 | GTCTTTCACCTTCATAGACCTTGAT | GTCCCCTTACACTTGGTATTGA |

Primer sequences for *mt-1, c-fos,* and *grp-78* were designed using Primer3 software (<http://fokker.wi.mit.edu/primer3/input.htm>).
