# Supplementary material for: Cadmium Induces Transcription Independently of Intracellular Calcium Mobilization
Source: PLoS One. 2011 Jun 9;6(6):e20542. doi: 10.1371/journal.pone.0020542 (PMC3111418; doi:10.1371/journal.pone.0020542)
Supplement: Table S2 — Functional gene grouping of human cAMP/calcium PCR Array. (DOCX) [file pone.0020542.s002.docx]

**Table S2**

**Functional gene grouping of human cAMP/ calcium PCR Array**

|  | 1 | 2 | 3 | 4 | 5 | 6 | 7 | 8 | 9 | 10 | 11 | 12 |
| --- | --- | --- | --- | --- | --- | --- | --- | --- | --- | --- | --- | --- |
| A | ADRB1 | AHR | AMD1 | AREG | ATF3 | BCL2 | BDNF | BRCA1 | CALB1 | CALB2 | CALM1 | CALR |
| B | CCNA1 | CCND1 | CDK5 | CDKN2B | CGA | CHGA | CNN1 | CREB1 | CREM | CTF1 | CYR61 | DDIT3 |
| C | DUSP1 | EGR1 | EGR2 | ENO2 | FGF6 | FOS | FOSB | GCG | GEM | GIPR | HK2 | HSPA4 |
| D | HSPA5 | IL2 | IL6 | INHBA | JUNB | JUND | KCNA5 | LDHA | MAF | MIF | NCAM1 | NF1 |
| E | NOS2A | NPY | NR4A2 | PCK2 | PCNA | PENK | PER1 | PLAT | PLN | PMAIP1 | POU1F1 | POU2AF1 |
| F | PPP1R15A | PPP2CA | PRKAR1A | PRL | PTGS2 | RB1 | S100A12 | S100A6 | S100G | SCG2 | SGK1 | SLC18A1 |
| G | SOD2 | SRF | SST | SSTR2 | STAT3 | TACR1 | TGFB3 | TH | THBS1 | TNF | VCL | VIP |
| H | B2M | HPRT1 | RPL13A | GAPDH | ACTB | HGDC | RTC | RTC | RTC | PPC | PPC | PPC |

Fill color: red = genes whose promoters contain SRE or SRE-like elements

white = genes whose promoters contain CRE elements

green = genes whose promoters contain an SRE and CRE element

blue = genes whose promoters contain other calcium responsive elements

pink = housekeeping genes.
